# Primary Retrobulbar Leiomyosarcoma in a Dog: A Case Report

**DOI:** 10.3390/vetsci7040145

**Published:** 2020-09-27

**Authors:** Joong-Hyun Song, Do-Hyeon Yu, Dong-In Jung

**Affiliations:** Institute of Animal Medicine, College of Veterinary Medicine, Gyeongsang National University, Jinju 52828, Korea; wndgus611@gmail.com (J.-H.S.); yudh@gnu.ac.kr (D.-H.Y.)

**Keywords:** CT, dogs, immunohistochemistry, leiomyosarcoma, MRI, retrobulbar tumor

## Abstract

A 2-year-old female Mongrel dog weighing 3.12 kg presented with a 2-month history of progressive exophthalmos of the left eye and periorbital swelling. Fine-needle aspiration cytology of the affected tissue revealed atypical cells of suspected malignant mesenchymal tumor origin. Computed tomography and magnetic resonance imaging revealed an ill-demarcated soft tissue mass in the left retrobulbar space extending into the nasal cavity and into the frontal lobes of the brain with destruction of the adjacent cribriform plate and the basisphenoid bone. Histopathological features of the tumor were consistent with the diagnosis of undifferentiated sarcoma. The tumor cells were immunoreactive for vimentin, smooth muscle actin, and desmin and negative for S100. These findings were mostly consistent with leiomyosarcoma arising from the smooth muscle on the retrobulbar tissues. Primary retrobulbar leiomyosarcoma is an extremely rare tumor in dogs. To expand our knowledge of retrobulbar leiomyosarcoma in dogs, we have described its clinical, diagnostic imaging, histopathological, and immunohistochemical characteristics in a dog.

## 1. Introduction

A retrobulbar tumor refers to any tumor situated in the retrobulbar space, which is the area located behind the globe of the eye. Retrobulbar tumors rarely occur in humans and in veterinary species. The majority of the retrobulbar tumors are primary and malignant, which often leads to the affected animals being euthanized without further investigation. Unfortunately, even with proper interventional treatment, including surgery, radiotherapy, or chemotherapy, the prognosis of retrobulbar tumors is extremely poor [1]. Unilateral exophthalmos is the most prominent clinical feature of retrobulbar tumors. The eye globe on the affected side is usually pushed out of its axis due to the location and the space-occupying property of the lesion.

Leiomyosarcoma is a malignant smooth muscle tumor of mesenchymal origin that can occur in any region where smooth muscle cells are present. Leiomyosarcoma in dogs is known to arise primarily from the gastrointestinal tract, spleen, and liver [2]. Leiomyosarcoma rarely involves the urinary bladder, uterus, vagina, vascular tissue, peritoneum, and other deep soft tissues. Only one case of retrobulbar leiomyosarcoma in a dog has been reported in the veterinary literature to date. However, there is no detailed description about the medical records of the animal [3].

To broaden our knowledge of retrobulbar leiomyosarcoma in dogs, we have described a rare case of retrobulbar leiomyosarcoma and its clinical, diagnostic imaging, histopathological, and immunohistochemical characteristics in a dog.

## 2. Case Presentation

**Figure 1:**
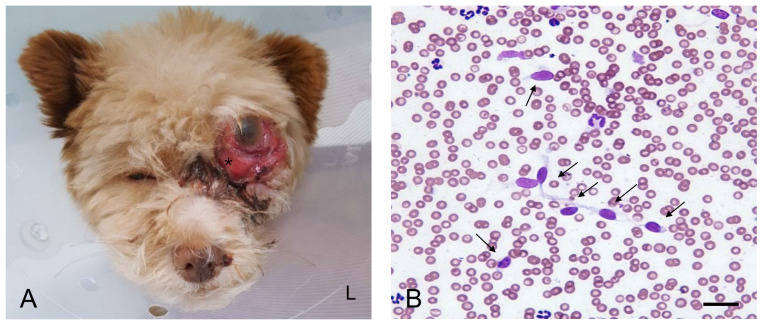
Gross and cytological findings of a retrobulbar mass in the present case. (**A**) A retrobulbar mass (asterisk) in a dog resulted in exophthalmos, superior deviation of the globe of the left eye off its axis, and exposure. (**B**) Fine-needle aspiration cytology of the mass revealed atypical cells (arrows) of suspected malignant mesenchymal tumor origin (Diff-Quik stain, 400×; scale bar = 20 μm). L: left.

A 2-year-old female Mongrel dog weighing 3.12 kg presented with a 2-month history of progressive exophthalmos and conjunctival hyperemia in the left eye. The owner noticed an actively growing mass located behind the globe OS. Globe luxation occurred when the globe was displaced anteriorly beyond the orbital rim. On admission to the hospital, the mass resulted in dorsal deviation of the left globe (Figure 1A). The periorbital region was ulcerated and swollen. The dog experienced pain on palpation in the left periorbital region. The affected eye was non-visual. The pupil was enlarged with no response to light stimuli. The neurological examination was unremarkable. The dog was generally healthy until the appearance of the mass and had been fully vaccinated and dewormed with no history of trauma. Except ocular abnormalities, no other abnormalities were noted on complete physical examination. Complete blood cell count analysis (ProCyte Dx, IDEXX Laboratories, ME, USA) revealed mild non-regenerative anemia (hematocrit: 35.5%, reference range: 37.3–61.7) and leukocytosis (30.96 × 10^9^ cells/L, reference range: 5.05–16.76 cells/L) secondary to neutrophilia (25.84 × 10^9^ cells/L, reference range: 2.95–11.64 cells/L). The results of serum biochemical analysis (Catalyst One Chemistry Analyzer, IDEXX Laboratories Inc., ME, USA) were unremarkable. Survey radiographs of the thorax and the abdomen were categorized as normal.

Fine-needle aspiration cytology (FNAC) of the retrobulbar mass revealed atypical cells of suspected malignant mesenchymal tumor origin (Figure 1B). Scattered cells were exfoliated individually and appeared spindle-shaped or stellate with indistinct cytoplasmic borders. Oval nuclei, anisokaryosis, high nuclear-to-cytoplasmic ratio, multiple and variable-shaped nucleoli, and marked cellular pleomorphism suggested a malignant mesenchymal neoplasm.

**Figure 2:**
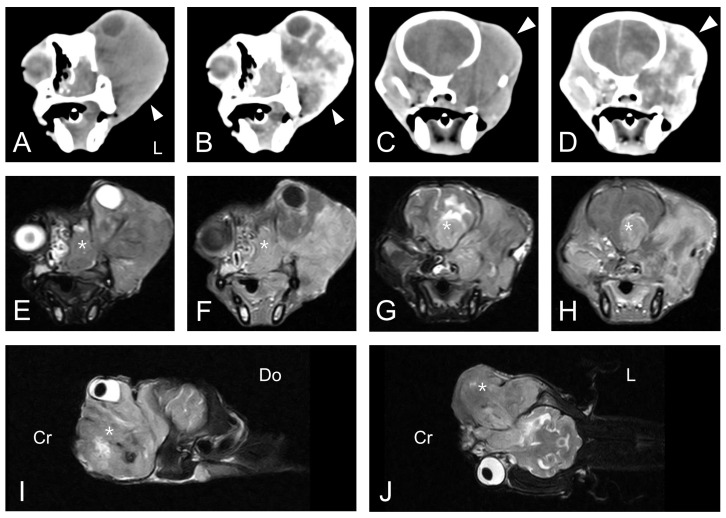
Transverse-plane computed tomography (CT) images (**A**–**D**), magnetic resonance images (MRI) (**E**–**H**) at the level of the eye globes and the brain, and left parasagittal (**I**) and dorsal plane (**J**) MRI are depicted. Both pre-contrast (**A**,**C**) and post-contrast (**B**,**D**) CT images demonstrated an ill-defined, heterogeneous, contrast-enhanced soft tissue mass (arrowhead) extending from the left nasal cavity to the left pterygopalatine fossa. Sequential MRI revealed an equivalent lesion that was hyperintense on T2W (**E**,**G**,**I**,**J**) and irregularly enhanced on CET1W images (**F**,**H**). The space-occupying mass (asterisk) with vasogenic edema caused significant mass effect and midline shift to the affected side of the brain. L, left; Cr, cranial; Do, dorsal.

The dog underwent computed tomography (CT) and magnetic resonance imaging (MRI). CT was performed using the Somatom Emotion Duo scan system (Siemens Medical Systems, Munich, Germany). Pre-contrast and post-contrast transverse images with a 3-mm slice thickness were obtained. All post-contrast images were obtained following the injection of intravenous iohexol (Omnipaque 300; GE-Healthcare, Little Chalfont, United Kingdom) at a dose of 0.9 g I/kg body weight. MRI was performed using the APERTO 0.4 T scanner (Hitachi Medical Corporation, Tokyo, Japan). T1-weighted (T1W) images, T2-weighted (T2W) images, fluid-attenuated inversion recovery (FLAIR) images, and contrast-enhanced T1-weighted (CET1W) images were obtained from the MRI scan. CET1W images were obtained after intravenous injection of gadolinium EDTA (Omniscan; GE-Healthcare, Little Chalfont, United Kingdom) at a dose of 0.20 mmol/kg body weight. CT scan of the head revealed an ill-defined, heterogeneous, isoattenuating to hyperattenuating, contrast-enhanced soft tissue mass in the left orbit with superior displacement of the left globe (Figure 2A–D). The mass completely occupied the posterior aspect of the lateral nasal cavity and the left pterygopalatine fossa. It also exhibited invasion of the skull base with lysis of the adjacent bone, including the maxilla, cribriform plate, and basisphenoid. Whole-body CT scan could not find any evidence of an extraocular primary tumor or distant metastases. An MRI scan at the same anatomic level as the CT scan revealed an equivalent mass in the left retrobulbar space extending into the nasal cavity and into the frontal lobes of the brain with destruction of the adjacent cribriform plate and the basisphenoid bone (Figure 2E–J). The lesion was hyperintense on T2W and FLAIR images, hypointense on T1W images, and irregularly enhanced on CET1W images. The space-occupying mass with vasogenic edema caused a significant mass effect and midline shift to the affected side of the brain.

**Figure 3:**
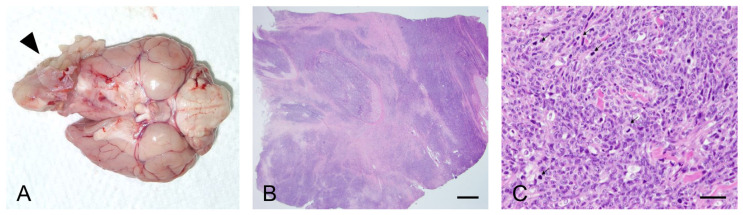
Gross and histopathological examination of the retrobulbar mass after necropsy. (**A**) Gross photograph of the exenterated brain (ventral aspect) specimen showed a mass (arrowhead) from the retrobulbar tissue infiltrating the cranial aspect of the left hemisphere. (**B**,**C**) Histopathological section of the mass (hematoxylin and eosin, B, ×12.5, scale bar = 250 μm; C, ×400, scale bar = 50 μm) revealed dense and poorly differentiated malignant spindle-shaped cells with frequent mitotic figures (arrows).

Forty days after admission, the dog was euthanized at the owner’s request. We performed gross and histopathological examination of the retrobulbar mass. Examination of the orbit revealed a large, poorly demarcated, and protruding soft tissue mass located behind the left eye globe showing extensive invasion of the ipsilateral nasal cavity and the cerebral hemisphere with destruction of the surrounding tissue (Figure 3A). Histopathological examination revealed a mass composed of dense poorly differentiated malignant spindle-shaped cells (Figure 3B,C). Marked anisocytosis and anisokaryosis were observed with 43 mitotic figures in 10 high-power fields. Microscopic infiltration of the tumor cells into the intratumoral lymphatic vessels and into the adjacent brain tissue was also observed. Although the histopathological findings were consistent with the diagnosis of sarcoma, the highly undifferentiated nature of the tumor made it difficult to characterize the specific origin of the tumor.

**Figure 4:**
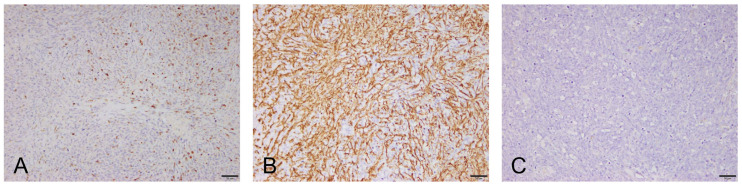
The figure depicts immunohistochemical staining of the tumor cells (counterstained with hematoxylin, 100×, scale bar = 50 μm). The tumor cells were positive for desmin (**A**) and smooth muscle actin (**B**) and negative for S100 (**C**).

To further characterize the tumor, immunohistochemical (IHC) analysis with vimentin, desmin, smooth muscle actin, and S100 was performed. Canine normal uterus tissue, including the smooth muscle layer (for vimentin, SMA, and desmin) and canine normal brain tissue (for S100), were used as the positive control. Negative controls were performed by omitting the primary antibody. The tumor cells were positive for vimentin (not recorded), desmin, and smooth muscle actin and negative for S100 (Figure 4). Immunohistochemical characteristics of the tumor cells were most consistent with leiomyosarcoma arising from the smooth muscle on the retrobulbar tissues (retrobulbar leiomyosarcoma).

## 3. Discussion

Orbital leiomyosarcoma can arise as a primary tumor from the vascular tissue or the smooth muscular tissue of the orbital component. Only one case of orbital (retrobulbar) leiomyosarcoma in dogs has been reported previously without a detailed description of the animal [3]. Similarly, cases of orbital leiomyosarcoma have rarely been reported in humans [4]. Despite the limited number of case studies, this tumor is characterized by distant metastasis and local invasion into the surrounding tissue, including the eye, nasal cavity, retrobulbar area, and cranium, due to the aggressive nature of the tumor. It is also speculated that older females are predominantly affected by orbital leiomyosarcoma, as observed in leiomyosarcoma in dogs [2]. Similar to the previous report, the dog in the present case was female, but not old, and the tumor exhibited extensive invasion into the surrounding structures of the eye, nasal cavity, and brain.

Retrobulbar tumors are extremely rare among periorbital tumors in humans as well as in veterinary species. Due to the location of the tumor, it is difficult to detect in the early stages. Therefore, it is more difficult to manage than other periorbital tumors. The clinical signs are also obscure and insidiously progressive. Unilateral exophthalmos is the most prominent clinical sign and manifestations secondary to exophthalmos can initially be confused with intraocular diseases, such as glaucoma [1]. Exophthalmos was also observed in the present case along with conjunctival hyperemia in the affected eye globe. Thus, the owner was unable to suspect a retrobulbar mass until after an observation period of 2 months. The dog was judged to be in a fairly late stage of the disease. Thus, the dog was euthanized without any therapeutic trials at the owner’s request.

Leiomyosarcomas are known to be aggressively invasive but slow-growing sarcomas. The reported median survival time for leiomyosarcomas in other sites than the retrobulbar space is 10 months (ranging from 1 month to 7 years) [2]. Tumor growth in the present case was markedly faster than that usually observed in leiomyosarcomas (survival time of 40 days). In a previously reported case of retrobulbar leiomyosarcoma, the canine showed a relatively short survival time (137 days) even after total orbitectomy at the initial diagnosis [3]. Hence, although additional cases need to be studied, it is possible that the primary retrobulbar leiomyosarcoma in dogs is intrinsically more aggressive than leiomyosarcomas at other locations.

The diagnosis of tumors that are likely to originate from undifferentiated mesenchymal cells can be challenging in veterinary practice. It requires many step-up procedures and is associated with considerable financial burden. Furthermore, the regional anatomical features of retrobulbar tumors present several limitations in the entire diagnostic process. In the present case, the diagnosis of retrobulbar leiomyosarcoma was established based on a detailed analysis of clinical features, diagnostic imaging features, and microscopic information. Meticulous gross examination led to suspected space-occupying structural changes behind the globe of the left eye. Microscopic analysis of FNAC revealed the possibility of a sarcoma. CT and MRI were performed to confirm the presence of retrobulbar mass and to delineate its extent and metastatic potential. The diagnosis was eventually confirmed by histopathology and IHC examination of the exenterated mass. IHC is very useful in differentiating a specific type of sarcoma from other tumors of mesenchymal origin. Panels of IHC markers, such as smooth muscle actin, desmin, vimentin, S100 protein, HMB45, CD34, and CD117, are often used in human oncology for leiomyosarcoma [5]. Leiomyosarcomas exhibit positive immunoreactivity to smooth muscle actin and desmin. IHC for the S100 protein marker can be used to rule out neurogenic tumors [6]. Immunoreactivity to desmin confirms the myogenic origin of tumors. It is commonly detected in smooth muscle and skeletal muscle tumors. Smooth muscle actin is a smooth muscle-specific antibody that can distinguish leiomyosarcomas from other sarcomas of muscle cell origin, such as rhabdomyosarcomas [7]. Orbital rhabdomyosarcoma has been relatively well described in a previous retrospective study in canines. It shares many clinical similarities with the present case of retrobulbar leiomyosarcoma [8]. Therefore, IHC staining using antibodies against smooth muscle actin is essential for distinguishing periorbital leiomyosarcomas from other types of undifferentiated myogenic sarcomas.

Leiomyosarcoma of the present report occurred in a patient at a very young age. In human oncology, it is well known that soft-tissue sarcomas, including leiomyosarcoma, are associated with germline mutations of the TP53 tumor-suppressor gene in children and young adults (Li–Fraumeni syndrome) [9]. Although there is no research considering the onset age of tumors, TP53 gene mutation has also been demonstrated in some soft-tissue sarcomas of dogs with bad prognosis [10,11]. Regarding these aspects, there is a possibility that leiomyosarcoma in this patient at an early age is associated with mutation of the TP53 gene, but this was beyond the scope of the present study. Thus, further investigation will be needed to clarify the association of TP53 gene mutation with soft-tissue sarcomas in younger canine patients.

Due to the aggressive behavior of leiomyosarcoma in the present case, early diagnosis would have resulted in a more favorable prognosis. Previous data support the suggestion that aggressive surgical excision with total orbitectomy can be used to effectively manage invasive orbital tumors [3]. If the tumor had not invaded a vital neighboring organ and distant metastasis had not been identified, surgical resection was a reasonable option. Thus, retrobulbar malignant tumors should be highly suspected in patients with abrupt exophthalmos and clinical signs secondary to exophthalmos for early intervention.

## 4. Conclusions

The present report describes a case of primary retrobulbar leiomyosarcoma in a canine. Primary retrobulbar leiomyosarcoma is an extremely rare tumor in dogs. The findings in the present case indicate that leiomyosarcoma of the retrobulbar area in canines can occur at a relatively young age and appears to be intrinsically more aggressive than leiomyosarcomas at other locations. CT and MRI are useful in the detection of lesions and in delineating their extent. Detailed histopathology and IHC analysis are highly valuable in diagnosis and in distinguishing primary leiomyosarcomas from other tumors of mesenchymal cell origin. Despite the low prevalence of retrobulbar leiomyosarcoma in dogs, veterinary clinicians should consider leiomyosarcoma in the differential diagnosis of a retrobulbar mass.
